# Dietary Specialization during the Evolution of Western Eurasian Hominoids and the Extinction of European Great Apes

**DOI:** 10.1371/journal.pone.0097442

**Published:** 2014-05-21

**Authors:** Daniel DeMiguel, David M. Alba, Salvador Moyà-Solà

**Affiliations:** 1 Institut Català de Paleontologia Miquel Crusafont, Universitat Autònoma de Barcelona, Barcelona, Spain; 2 Dipartimento di Scienze della Terra, Università di Torino, Torino, Italy; 3 ICREA at Institut Català de Paleontologia Miquel Crusafont and Unitat d’Antropologia Biològica (Dept. BABVE), Universitat Autònoma de Barcelona, Barcelona, Spain; University of Florence, Italy

## Abstract

Given the central adaptive role of diet, paleodietary inference is essential for understanding the relationship between evolutionary and paleoenvironmental change. Here we rely on dental microwear analysis to investigate the role of dietary specialization in the diversification and extinction of Miocene hominoids from Western Eurasian between 14 and 7 Ma. New microwear results for five extinct taxa are analyzed together with previous data for other Western Eurasian genera. Except *Pierolapithecus* (that resembles hard-object feeders) and *Oreopithecus* (a soft-frugivore probably foraging opportunistically on other foods), most of the extinct taxa lack clear extant dietary analogues. They display some degee of sclerocarpy, which is most clearly expressed in *Griphopithecus* and *Ouranopithecus* (adapted to more open and arid environments), whereas *Anoiapithecus*, *Dryopithecus* and, especially, *Hispanopithecus* species apparently relied more strongly on soft-frugivory. Thus, contrasting with the prevailing sclerocarpic condition at the beginning of the Eurasian hominoid radiation, soft- and mixed-frugivory coexisted with hard-object feeding in the Late Miocene. Therefore, despite a climatic trend towards cooling and increased seasonality, a progressive dietary diversification would have occurred (probably due to competitive exclusion and increased environmental heterogeneity), although strict folivory did not evolve. Overall, our analyses support the view that the same dietary specializations that enabled Western Eurasian hominoids to face progressive climatic deterioration were the main factor ultimately leading to their extinction when more drastic paleoenvironmental changes took place.

## Introduction

### Dietary Adaptation and the Hominoid Radiation in Western Eurasia

After an initial radiation in Africa during the Early to Middle Miocene [1], hominoids dispersed into Eurasia, where they diversified into multiple great ape genera from ca. 14 Ma onwards [2]–[6]. Available data suggest that vicariance and parallel evolution played a significant role in the Eurasian hominoid radiation, with dryopithecines diversifying in Europe, pongines in Asia, and maybe hominines in Africa [3], [4]. Dietary adaptations have long been regarded as very significant for understanding the dispersal of hominoids from Africa into Eurasia and their subsequent radiation [4], [6]–[8]. Paleodietary inference is thus paramount for understanding how fossil great apes adapted to changing environmental conditions through time. Unlike in hominins, however, comparatively little dietary research has focused on fossil great apes from Eurasia [7]–[15]. Previous results suggest that a considerable dietary diversity was present in the Late Miocene, and that such diversification might have taken place during the Middle and Late Miocene [7].

Previous dental microwear analyses were based on a wide array of extinct hominoids from the Miocene of Western Eurasia [10]–[12]: *Griphopithecus alpani* [16], [17] from the early Middle Miocene (MN6, ca. 14.9–13.7 Ma) [3] of Turkey; *Hispanopithecus* (*Rudapithecus*) *hungaricus* [18], [19] from the Late Miocene (MN9, ca. 10.0–9.8 Ma) [3] of Hungary; *Ouranopithecus macedoniensis* [20]–[22] from the Late Miocene (MN10, ca. 9.7–9.0 Ma) [3] of Greece; *Oreopithecus bambolii* [23]–[25] from the Late Miocene (MN12, ca. 8.3–6.7 Ma) [26] of Italy; and *Hispanopithecus crusafonti* (MN9, ca. 10.4–10.0 Ma) and *Hispanopithecus laietanus* (MN9, ca. 11.1–9.5 Ma) [4] from Spain [3]. Results based on these taxa [10]–[12] suggested that, from the hard-object feeding plesiomorphic condition displayed by *G. alpani* [12], a progressive dietary diversification and specialization would have taken place through time—with *Hispanopithecus* spp. being inferred as a frugivore [10], [11], *Ou. macedoniensis* as a hard-object specialist [10], and *O*. *bambolii* as an extreme folivore [10].

These analyses had, however, a significant gap, because late Middle Miocene taxa were not included due to the scarcity of fossil specimens from this time span. This situation, however, drastically changed during the last decade thanks to continued and extensive fieldwork in the late Middle Miocene local stratigraphic series of Abocador de Can Mata in els Hostalets de Pierola (Vallès-Penedès Basin) [4], [27], [28]. The material recovered there has shown an unprecedented diversity of Middle Miocene dryopithecines in Western Europe [3], [4], with two new genera and species (*Pierolapithecus catalaunicus* [29] and *Anoiapithecus brevirostris* [30]) being described, and new material of *Dryopithecus fontani* [31] being recovered. Similarly, excavations at several Late Miocene localities of the same basin have led to the recovery of new dental remains of *Hispanopithecus laietanus*, which further confirm the distinction of this species from *Hispanopithecus crusafonti*, recorded at slightly older localities [4]. Additional paleodietary data are therefore required for these taxa in order to better understand the hominoid radiation in Western Eurasia from a dietary viewpoint.

**Figure 1:**
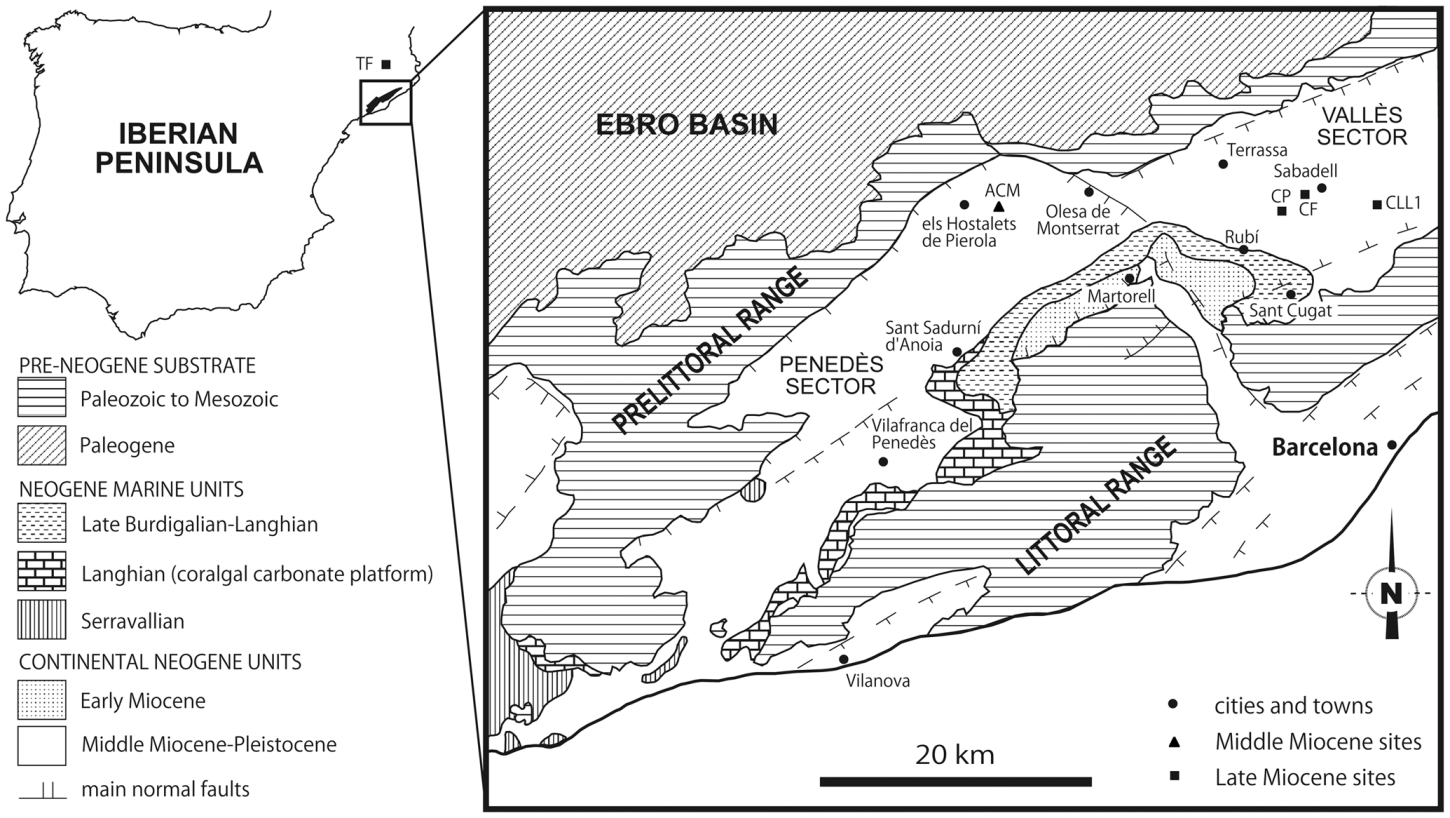
Schematic geological map. Square showing the location of the hominoid-bearing localities that have delivered dental remains studied in this paper, with emphasis on those from the Vallès-Penedès Basin (NE Spain).

Here we report new dental microwear analyses for five hominoids from the Iberian Peninsula (Figure 1), ranging in age from 12.3–12.2 to 9.7 Ma [3], [4]. Together with previous results for other Miocene hominoids from elsewhere in Europe and Turkey [10]–[12] (see Table S1), these data allow us to re-evaluate dietary diversification during great-ape evolution in Western Eurasia (ca. 14 to 7 Ma) in the light of paleoenvironmental changes.

### Paleodietary Inference

Among the various methods of paleodietary inference in primates, dental gross morphology and ultrastructure are useful because teeth are adapted for food processing [32]. Occlusal morphology, which can be quantified by means of shearing crest analysis [9], [11], [33], offers some clues because folivorous apes possess longer shearing crests on molars than frugivorous ones. However, shearing crest quotients are highly dependent on the particular group being analyzed and the baseline used as a reference for comparison [11], [33], so that they might be potentially biased when applied to extinct taxa [34]. Enamel thickness is also generally considered to reflect dietary adaptations to some degree, given the relationship between hard-object feeding and thick enamel [7], [8], [35]–[37]. However, enamel thickness is heavily influenced by phylogenetic constraints [38] and there is no threshold value for distinguishing hard-object feeders on this basis alone [39], [40], so that overall there is no direct relationship between enamel thickness and diet. All these morphology-based approaches reflect dietary adaptation as well as phylogenetic constraints, and hence probably are more informative about what extinct taxa were able to eat than about what they actually ate [41].

Non-morphological methods of paleodietary reconstruction, based on either dental microwear or stable isotope geochemistry, provide more direct information on the properties of the foods consumed independently from adaptation [41], [42]. Geochemical methods, such as stable carbon isotope ratios derived from fossil tooth enamel, are based on the fact that hominoids that consumed grasses and sedges have higher ^13^C levels than those that fed on fruits and other plants [41]–[46]. These methods are based on the carbon isotopic distinction between C_3_ and C_4_ photosynthetic pathways. However, C_4_ plants (grasses) did not globally expand until the Late Miocene (ca. 8-6 Ma) [47], [48], being present in Eurasia only from 9.4 Ma onwards [49]—i.e., after the extinction of most of the Western Eurasian hominoids studied in this paper. Moreover, isotopic analysis of tooth enamel implies invasive sampling techniques, which are not advisable given the small available dental samples for most of the studied taxa. Dental microwear analysis similarly provides direct evidence on the type of food items consumed by a particular fossil individual, but unlike isotopic methods it is a non-invasive technique, which relies on the microscopic traces left by foods on the enamel surface [10], [15], [50].

Dental microwear analysis is thus one of the most powerful methods for inferring dietary behavior in extinct taxa, being based on the strong and consistent association between dental microscopic patterns and the physical properties of the chewed foods [15], [50]. While both occlusal morphology and enamel thickness might provide important clues as to what types of food a particular taxon was adapted to consume, microwear features directly reflect what an animal actually ate just prior to death [51]. Hence, since the early 1980s dental microwear analysis has been extensively applied to early hominins and other fossil primates in order to try to determinate their dietary behavior (and seasonal changes thereof), as well as tooth use and masticatory jaw movements [12]. Dental microwear provides direct information on the type of food items consumed shortly prior to an individual's death (days, weeks or months, depending on the nature of the foods being masticated)—a phenomenon referred to as the “Last Supper effect” [52]. The physical properties of both the food items (especially phytoliths from plant taxa) and of exogenous grit (abrasive dirt) being ingested during feeding influence dental microwear patterns. Based on both research in the wild [53] and experimental studies [54], [55], some authors have contended that exogenous grit plays an important (or even a primary) role in dental microwear genesis—which might explain why some species have broadly-similar microwear patterns in spite of marked dietary differences [56]. Further experimental research is undoubtedly necessary to better understand the mechanisms responsible of microwear formation, and particularly to determine the role of exogenous grit as a causative agent. However, various studies support the view that food item properties are the main factor determining microwear genesis [57]–[59]. Thus, work on primates and other mammals has shown a strong relationship between dental microwear features and the types of food consumed, as indicated by different taxa from comparable sites, which exhibit microwear differences that are consistent with their contrasting diets [59]–[61].

Dental microwear texture analysis [62]–[65] was recently introduced as an alternative technique to more traditional methods of microwear analysis, being based on 3D surface data and scale-sensitive fractal analysis. Unlike the traditional method, texture analysis does not require the identification of individual features and the analysis is automated—thus being less affected by interobserver error and much less time consuming [51], [62]. Contrary to such advantages, microwear texture analysis is a much more costly alternative, because it relies on white-light scanning confocal microscope instead of 2D micrographs taken with a standard Scanning Electron Microscope (SEM). Texture analysis was introduced to increase repeatability and avoid interobserver error [62], but error studies of traditional microwear quantification techniques show that high errors are found only when different methodologies are employed [66]. As long as a consistent technique is employed, such as that offered by the Microware software package, a common microwear database derived by different researchers can be consistently employed [66]. In this sense, using the traditional microwear analysis approach offers the advantage that our new results can be analyzed together with those derived by previous researches for both the extant comparative sample and other extinct hominoids. Whereas traditional microwear data are available for some Western Eurasian hominoids [10], [12], no microwear texture data have been thus far published for Miocene apes. As a result, the more traditional approach to microwear analysis followed in this work is still currently used by various researchers [34], [67], [68].

## Methods and Materials

### Dental Microwear

Micrographs of the occlusal enamel surfaces of the investigated teeth were taken with an environmental SEM (FEI Quanta 200) at the Serveis Cientificotècnics of the Universitat de Barcelona (Spain), on “Phase II” crushing/grinding facets (9, 10n and x) [20], [69]. The standard procedure described in ref. [50] was employed, including 500× magnification and 200 dpi micrograph resolution, in secondary emissions mode and a 20 kV voltage. To avoid interobserver error, an area of 0.02 mm^2^ [11], [70] was analyzed with Microware 4.02^©^ software by a single author (DDM). Some of the examined specimens showed extensive microscopic damage and were therefore discarded.

Two main microwear features (pits and striations) were distinguished [15], [50], [60]. Pits are microwear scars that are circular or subcircular in outline. Scratches, in turn, are elongated microfeatures with straight, parallel sides. In this study, pits and scratches were directly categorized by following an arbitrarily-set length to width ratio of 4:1 [50]. The three standard variables customarily quantified in dental microwear analyses were employed [15], [50], [71]: (1) Percentage of pits (%), i.e., the proportion of pits relative to the total number of microwear features; (2) breadth of striations (in µm); and (3) breadth of pits (in µm). Previous studies have shown that the relative proportion between pits and scratches enables the distinction between frugivores, folivores and hard-object feeders [10], [12], [15], [34], [71], [72]. Thus, although this is a continuous variable across dietary categories, there is a strong and significant positive correlation between the prevalence of pits and the consumption of hard, brittle foods (such as nuts), as well as between higher scratch frequencies and the consumption of tough items (such as leaves and softer fruits). Microwear feature size is also valuable for further characterizing diets, especially when combined with pitting incidence in multivariate analyses [34]. Moreover, striation breadth has been related to the ratio of exogenous grit versus phytoliths consumed in incisors [73], although this relationship remains to be tested in molar microwear.

### Studied Sample

We studied 15 upper and lower molars of the following Miocene hominoids (Table 1 and 2): *Pierolapithecus catalaunicus* from ACM/BCV1 (IPS21350) [29], *Anoiapithecus brevirostris* from ACM/C3-Aj (IPS41712 and IPS43000) [30] and ACM/C1-E* (IPS35027) [74], *Dryopithecus fontani* from ACM/C3-Ae (IPS35026) [31]; *Hispanopithecus* (*Hispanopithecus*) *crusafonti* from CP1 (IPS1820, IPS1818, IPS1812 and IPS1821) and TF (MGSB25314) [75], [76]; and *Hispanopithecus* (*Hispanopithecus*) *laietanus* from CF (IPS34753) [77] and CLL1 (IPS1763, IPS1788, IPS1797 and IPS1800) [76], [78], [79]. The taxonomy employed for hominoids follows ref. [4]. All specimens studied in this paper are housed at the Institut Català de Paleontologia Miquel Crusafont (Sabadell, Spain) and the Museu Geològic del Seminari de Barcelona (Barcelona, Spain). No permits were required for the described study. The ACM specimens come from the late Middle Miocene (MN7+8), whereas those from the remaining localities are Late Miocene (MN9) in age [3], [4]. To fully assess the available information and compensate for the small number of individuals in some cases, specimens of a single species from various localities were also analyzed together by using their average values for microwear variables. In particular, we combined specimens of *A*. *brevirostris* from ACM/C1-E* and ACM/C3-Aj, specimens from *H*. *crusafonti* from TF and CP1, and specimens of *H*. *laietanus* from CF and CLL1. This procedure is justified by the close geographic situation and age of these localities (Table 1, see also Figure 1, and Table S1).

**Table 1:** Summary results of the microwear analysis.

				Pits [%]			Pit breadth [µm]			Scratch breadth [µm]		
Taxon	Locality	Age [Ma]	N	Mean	SD	Range	Mean	SD	Range	Mean	SD	Range
*Pierolapithecus catalaunicus*	ACM/BCV1	11.93	1	42.50	—	—	9.59	—	—	3.79	—	—
*Anoiapithecus brevirostris*	ACM/C1-E*	12.3–12.2	1	19.23	—	—	3.05	—	—	2.87	—	—
*Anoiapithecus brevirostris*	ACM/C3-Aj	11.94	2	31.18	15.82	20.00–42.37	5.42	1.18	4.59–6.27	2.85	0.52	2.49–3.23
*Anoiapithecus brevirostris*	average	12.3–11.94	3	27.20	13.14	19.23–42.37	4.64	1.61	3.05–6.27	2.86	0.37	2.49–3.23
*Dryopithecus fontani*	ACM/C3-Ae	11.85	1	48.81	—	—	3.66	—	—	2.25	—	—
*Hispanopithecus crusafonti*	TF	10.4–10.0	1	51.39	—	—	4.24	—	—	2.60	—	—
*Hispanopithecus crusafonti*	CP1	10.4–10.0	4	33.43	5.63	27.84–41.17	4.78	1.37	3.78–6.79	2.37	0.12	2.25–2.54
*Hispanopithecus crusafonti*	average	10.4–10.0	5	37.02	9.40	27.84–51.39	4.67	1.21	3.78–6.79	2.42	0.14	2.25–2.60
*Hispanopithecus laietanus*	CF	10.0–9.7	1	34.61	—	—	6.77	—	—	2.44	—	—
*Hispanopithecus laietanus*	CLL1	9.72	4	29.33	6.30	21.66–37.07	5.08	0.84	4.26–6.25	2.59	0.47	2.07–3.22
*Hispanopithecus laietanus*	average	10.0–9.7	5	30.39	5.94	21.66–37.07	5.42	1.04	4.26–6.77	2.56	0.41	2.07–3.22

Abbreviations: N, sample size; SD, standard deviation.

Locality abbreviations: ACM, Abocador de Can Mata; BCV1, Barranc de Can Vila 1; C1, Cell 1; C3, Cell 3; CLL1, Can Llobateres 1; CP1, Can Poncic 1; TF, Teuleria del Firal.

**Table 2:** Results of the microwear analysis for all the analyzed specimens.

Taxon	Locality	Age (Ma)	Catalog No.	Tooth	% Pits	Pit Breadth	Scratch Breadth
*Pierolapithecus catalaunicus*	ACM/BCV1	11.93	IPS21350	LM1	42.50	9.59	3.79
*Anoiapithecus brevirostris*	ACM/C1-E*	12.3–12.2	IPS35027	LM1	19.23	3.05	2.87
*Anoiapithecus brevirostris*	ACM/C3-Aj	11.94	IPS41712	LM1	20.00	6.27	2.49
*Anoiapithecus brevirostris*	ACM/C3-Aj	11.94	IPS43000	Lm2	42.37	4.59	3.23
*Dryopithecus fontani*	ACM/C3-Ae	11.85	IPS35026	LM2	48.81	3.66	2.25
*Hispanopithecus crusafonti*	TF	10.4–10.0	MGSB25314	Lm2	51.39	4.24	2.60
*Hispanopithecus crusafonti*	CP1	10.4–10.0	IPS1820	LM2	33.33	4.07	2.37
*Hispanopithecus crusafonti*	CP1	10.4–10.0	IPS1818	LM1	31.37	6.79	2.54
*Hispanopithecus crusafonti*	CP1	10.4–10.0	IPS1812	RM2	27.84	3.78	2.25
*Hispanopithecus crusafonti*	CP1	10.4–10.0	IPS1821	RM2	41.17	4.46	2.32
*Hispanopithecus laietanus*	CF	10.0–9.7	IPS34753	m1	34.61	6.77	2.44
*Hispanopithecus laietanus*	CLL1	9.72	IPS1763	Rm1	21.66	4.26	2.46
*Hispanopithecus laietanus*	CLL1	9.72	IPS1788	RM1	28.78	4.97	2.60
*Hispanopithecus laietanus*	CLL1	9.72	IPS1797	Rm1	37.07	4.84	2.07
*Hispanopithecus laietanus*	CLL1	9.72	IPS1800	Lm3	29.82	6.25	3.22

Pit and scratch breadths reported in µm. Estimated ages taken from ref. [3].

Abbreviations: IPS, collections of the Institut Català de Paleontologia Miquel Crusafont; M, upper molar (followed by tooth position); m, lower molar (followed by tooth position); MGSB, Museu de Geologia del Seminari Conciliar de Barcelona; R, right; L, left. See locality abbreviations in Table 1.

### Comparative Samples

Our results were compared with those derived from previous authors [15], [60], [71] for a sample of 11 extant anthropoid primates with well-known diets (extant species samples consisting of 10 specimens, except that of *Papio cynocephalus*, which consists of 16). These studies were selected because they used a sufficiently similar technique to allow comparison with our results. As mentioned above, we analyzed our microwear results together with those previously published for other Western Eurasian Miocene hominoids, including: *Griphopithecus alpani* from Paşalar (MN6) [12], [13]; *Hispanopithecus hungaricus* from Rudabánya (MN9) [10], [11]; *Ouranopithecus macedoniensis* from Ravin de la Pluie, Xirochori and Nikiti (MN10) [10]; and *Oreopithecus bambolii* from Baccinello, Monte Bamboli and Ribolla (MN12) [10].

### Dietary Categories

Three extant dietary categories were employed by attributing each of the extant species to one of these groups defined a priori on the basis of published behavioral data [34]: (1) folivores (FOL); (2) frugivores/mixed feeders (FMF); and (3) hard-object feeders (HOF). Several species were subsumed into a single category of “frugivores/mixed feeders” [34], [80], because periods of fruit scarcity may impel many frugivorous primates to exploit alternative, non-preferred food sources (fallback foods), thereby resulting in a somewhat eclectic foraging strategy [34]. It should be also taken into account that the HOF category not only includes specialized hard-object feeders (*Lophocebus albigena* and *Cebus apella*) [60], [81], but also orangutans (*Pongo pygmaeus*), which are less specialized hard-object feeders but are not frugivores in a strict sense. All extant hominoids have a preference for ripe fruit, but the emphasis on leaves, soft fruits and hard food items various among the various species [7]—with orangutans consuming on average harder and unripe fruits more often than other great apes [13], [32], especially as fallback foods [37].

### Statistical Techniques

In order to offer insights into the dietary habits of species, hierarchical, complete-linkage (farthest neighbor method) cluster analyses based on Euclidean distances, and discriminant Canonical Variates Analyses (CVA) were used to analyse the extant and fossil data sets. Cluster analysis was intended to explore the similarities in microwear patterns between extant primates and extinct hominoids by using the above-mentioned three microwear variables. CVA, in turn, was intended to evaluate the reliability of these microwear variables for distinguishing between the various dietary categories defined for extant taxa, as well as to classify fossils to these categories. Extant taxa were thus included a priori in one of the three dietary categories described above, whereas the extinct hominoids were left unclassified and classified a posteriory on the basis of the classification probabilities derived by the analysis from Mahalanobis squared distances to extant group centroids. All statistical analyses were performed using the SPSS v. 11 statistical package.

### Technical Considerations

Like every paleobiological approach, dental microwear analysis has its particular drawbacks and limitations. When data derived from different researchers are combined into a single analysis, interobserver error in microwear features is a major concern that can complicate the interpretation of the results [66], [82]. This caveat applies to this study and should be borne in mind when interpreting our results. However, it is worth mentioning that several methodological precautions were adopted by us to minimize the error introduced. Thus, all the employed data were obtained through SEM imaging, which is less prone to error bias than other techniques such as light microscopy [83]. Moreover, the data used are based on procedures that, although not identical, are highly comparable because: (1) they were obtained using the same quantitative SEM-based technique; (2) microwear features were analyzed with a semiautomated image analysis procedures (primarily Microware 4.0), which results in lesser error rates [66]—with the exception of data taken from ref. [60], which employed traditional digitizer-based measurements; and (3) all the procedures employed followed standard methodological details, i.e., same wear facets selected for analysis, same instrumental settings (voltage, magnification and specimen detector), same micrograph resolution and analyzed surface, same measured microwear variables, etc. Finally, all the data measured in this analysis or taken from the literature were derived by experienced and highly trained microwear researchers, which diminishes the magnitude of error in microwear measurements [66], [84]. Although the use of these methodological precautions cannot fully remove the error bias, they provide a reasonable degree of interobserver consistency, thereby ensuring the comparability of the data employed.

## Results

### Microwear Features

**Figure 2:**
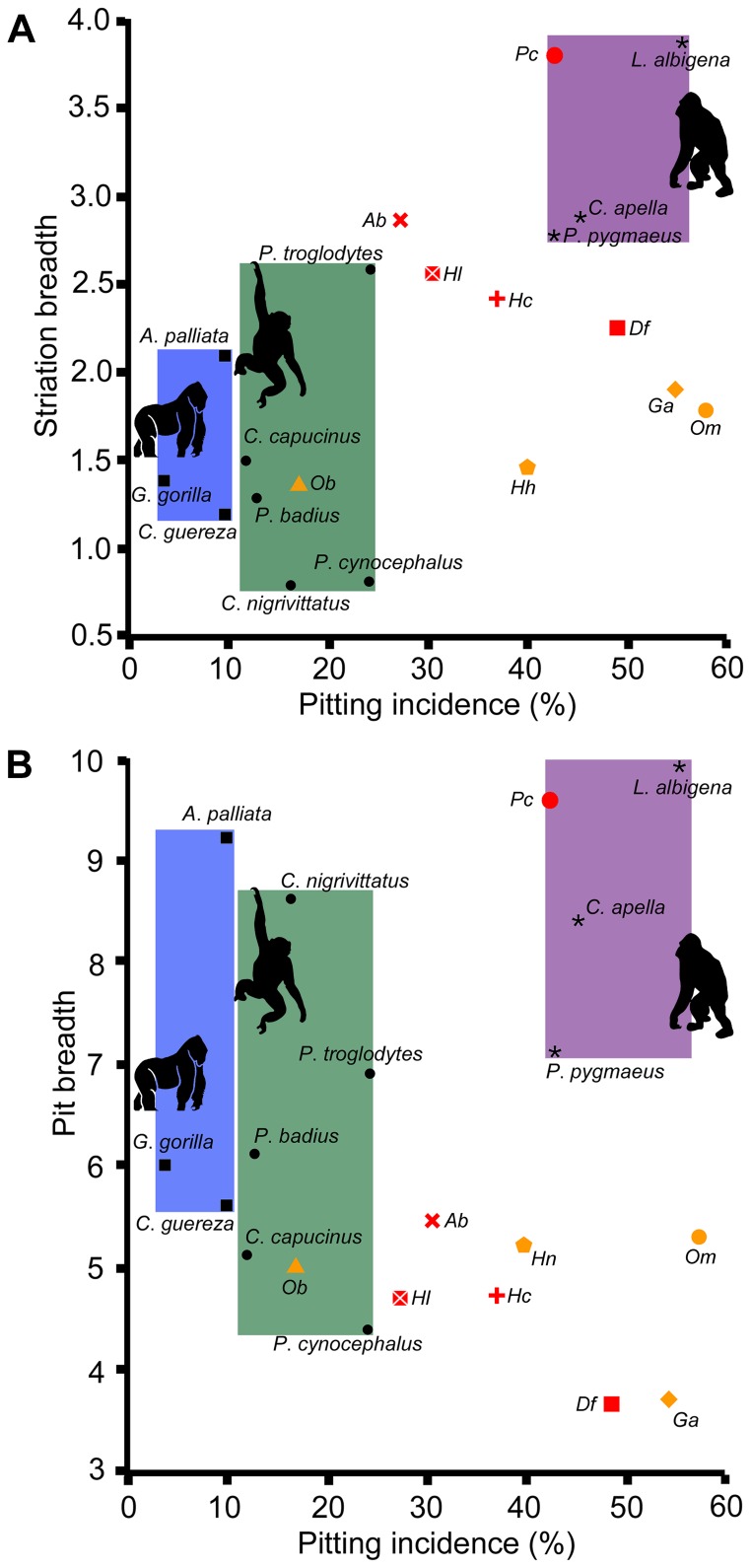
Bivariate plots of microwear feature breadth vs. pitting incidence. (**A**) Striation breadth and (**B**) pit breadth vs. pitting incidence based on species/locality means (Table 1). Fossil taxa abbreviations: *Ab*, *Anoiapithecus brevirostris*; *Df*, *Dryopithecus fontani*; *Ga*, *Griphopithecus alpani*; *Hc*, *Hispanopithecus crusafonti*; *Hh*, *Hispanopithecus hungaricus*; *Hl*, *Hispanopithecus laietanus*; *Ob*, *Oreopithecus bambolii*; *Om*, *Ouranopithecus macedoniensis*; *Pc*, *Pierolapithecus catalaunicus*. Different symbols are employed to distinguish each species. For species from multiple localities, only average values are shown (see individual values in Figure S1). The results derived in this study are depicted in red, whereas those taken from previous studies are shown in yellow. The polygons showing the variability of extant dietary categories are depicted in blue (folivores), green (mixed feeders/frugivores) and magenta (hard-object feeders).

Among the three analyzed variables (Table 1 and 2, and Figure 2 and S1), pitting incidence best distinguishes among dietary categories [50], [71], whereas microwear feature (especially striation) breadth allows to further refine paleodietary inferences [34]. With regard to pitting incidence, only *Pierolapithecus catalaunicus* and *Dryopithecus fontani* (represented by a single individual each) resemble extant HOF such as *Pongo pygmaeus* and *Lophocebus albigena*, which habitually consume hard and brittle items. Most of the remaining taxa are somewhat intermediate between *P*. *pygmaeus* and extant FMF such as *Pan troglodytes* and *Papio cynocephalus*. Although some differences between taxa/localities must partly reflect interindividual variation (Table 1 and 2, and Figure S1), the pitting incidences of all the hominoids from Spain suggest some degree of sclerocarpy. This is most clear in *P. catalaunicus*, which further displays wider striations—consistent with a preference for hard foods [34]—than in other extinct taxa, in the range of extant HOF and most closely resembling *L*. *albigena* (Figure 2 A). The remaining taxa (including *Hispanopithecus crusafonti* and *Hispanopithecus laietanus*) are intermediate between extant HOF and FMF when both pitting incidence and striation breadth are considered simultaneously (Figure 2 A). In contrast, none of the taxa overlaps with extant FOL for any of the studied variables.

Compared with other hominoids from Western Eurasia (Figure 2, Figure S1), only *Griphopithecus alpani* and *Ouranopithecus macedoniensis* surpass the pitting incidences of the hominoids from the Iberian Peninsula (and even that of extant HOF in some instances). The former, however, display much narrower microwear features than extant HOF and *P. catalaunicus*, thus overlapping with the remaining studied taxa (Figure 2 A, B). *Hispanopithecus hungaricus* overlaps to a large extent with other species of *Hispanopithecus* in the various microwear variables, thus being rather intermediate between FMF and HOF, whereas *Oreopithecus bambolii* uniquely falls within the range of extant FMF for most individuals.

### Multivariate Analyses

**Figure 3:**
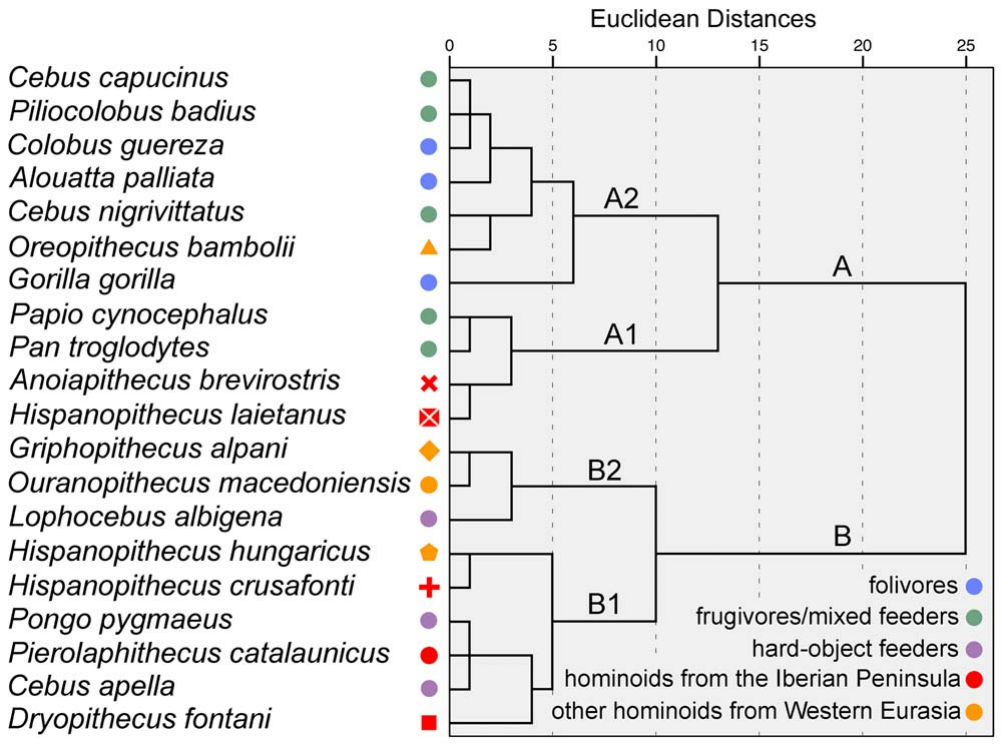
Cluster analysis based on dental microwear features. For species from multiple localities, only average values are shown (see individual values in Figure S2). Symbols and colors as in Figure 2.

A cluster analysis based on microwear fabrics (Figure 3) yields two main clusters separating FOL and FMF (cluster A) from HOF (cluster B). Among extinct hominoids, the average values of *A. brevirostris* and *H. laietanus* are grouped with the mixed feeder *P*. *cynocephalus* and the soft-fruit eater *P*. *troglodytes* in subcluster A1. The average of *O. bambolii* is in turn included in subcluster A2, together with the remaining extant frugivores and all FOL, which are characterized by lower pitting incidences. The remaining fossil hominoids clump together with HOF in cluster B, displaying higher pit percentages. Average values of *P. catalaunicus*, *D*. *fontani*, *H. crusafonti* and *H. hungaricus* are grouped with *P*. *pygmaeus* and *C*. *apella* in subcluster B1, whereas *G. alpani* and *Ou. macedoniensis* cluster with *L*. *albigena* in subcluster B2 (see Figure S3 for individual variation). Overall, the analyses indicate a hard-object feeding component for many of the fossil hominoids, with the exception of *A. brevirostris*, *H. laietanus* and *O. bambolii*, which show greater affinities with FMF. None of the taxa clusters with extant FOL.

**Figure 4:**
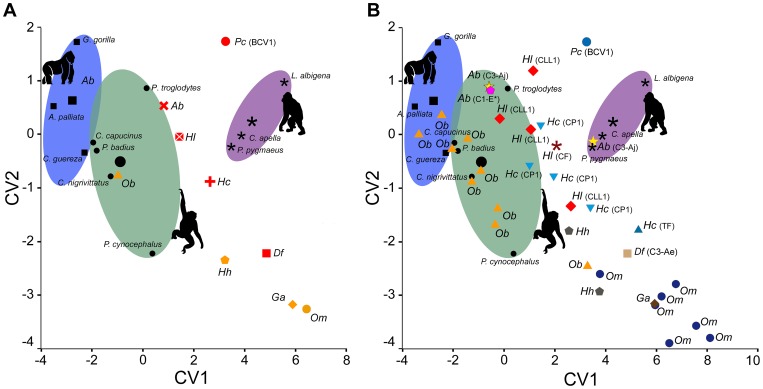
Results of the CVA based on three extant dietary categories and microwear variables. (A) Results based on mean/species locality data and (**B**) on individuals. Colored ellipses denote the morphospace defined by each extant group, black symbols extant taxa (group centroids by larger symbols). Symbols and colors in a as in Figure 2, whereas in B symbols and colors are different to show variability within fossil species.

The CVA (Figure 4 and Table 3; see also Table S2) confirms that the investigated variables provide a satisfactory dietary discrimination (100% of extant taxa correctly classified, 64% in cross-validation). CV1 separates HOF (positive values) from FMF and FOL (negative values) mostly on the basis of pitting incidence, whereas CV2, more influenced by scratch and pit breadths, does not enable a clear distinction among dietary categories. The discriminant analysis (Table S3) based on the CVA classifies most of the taxa as HOF, except the average values of *A. brevirostris*, *H. laietanus* and *O. bambolii*, which are classified as FMF (Figure 4 A). When individual classifications for extinct taxa are analyzed, *A. brevirostris*, *H. laietanus* and *H. crusafonti* display some variation in individual classifications between HOF and FMF, whereas several individuals of *O. bambolii* are classified as FOL or HOF instead of FMF (Figure 4 B, Tables 4 and S4). This fact reflects dietary diversity in some of the taxa, which indicates that caution is required when interpreting species (*P. catalaunicus* and *D. fontani*) represented by a single individual. However, based on classification probabilities (Table S4), *P. catalaunicus* falls within the variation of extant HOF, unlike *D. fontani*, *G. alpani* and *Ou. macedoniensis* (p<0.05). *Anoiapithecus brevirostris* and most individuals of *O. bambolii* similarly fit well with extant FMF. In contrast, the classification of *Hispanopithecus* species as either HOF or FMF is not consistent among individuals and not well supported for most of them, suggesting that they were truly intermediate between these categories.

**Table 3:** Results of the discriminant analysis per taxon/locality and for species average values.

Taxon	Locality	1st group	p	D^2^	2nd group	D^2^
*P*. *catalaunicus*	ACM/BCV1	HOF	0.192	3.296	FRU	22.219
*A. brevirostris*	ACM /C1-E*	FRU	0.375	1.961	FOL	5.012
*A.brevirostris*	ACM/C3-Aj	FRU	0.039	6.513	HOF	7.899
*A. brevirostris*	average	FRU	0.135	4.011	HOF	12.284
*D. fontani*	ACM/C3-Ae	HOF	0.041	6.403	FRU	36.059
*H. crusafonti*	TF	HOF	0.078	5.107	FRU	39.672
*H. crusafonti*	CP1	HOF	0.047	6.129	FRU	8.260
*H. crusafonti*	average	HOF	0.137	3.970	FRU	12.586
*H. laietanus*	CF	HOF	0.077	5.120	FRU	8.931
*H. laietanus*	CLL1	FRU	0.095	4.703	HOF	9.660
*H. laietanus*	average	FRU	0.067	5.410	HOF	8.612
*G. alpani*	Paşalar	HOF	0.001	14.400	FRU	53.895
*H. hungaricus*	Rudabánya	HOF	0.019	7.961	FRU	20.089
*O. bambolii*	Various	FRU	0.966	0.070	FOL	5.401
*Ou. macedoniensis*	Various	HOF	0.000	16.847	FRU	61.275

Abbreviations: D^2^, Squared Mahalanobis distance; FOL, folivores; FRU, frugivores/mixed-feeders; HOF, hard-object feeders; p, classification probability.

**Table 4:** Summary results for dietary classification (predicted group) of the fossil individuals studied in this paper according to the discriminant analysis based on microwear features.

**Hominoids from the Iberian Peninsula**			
**Taxon (locality)**	**Folivores**	**Frugivores**	**Hard-object feeders**
*P. catalaunicus* (ACM/BCV1)	0	0	1
*A. brevirostris* (ACM/C1-E*)	0	1	0
*A. brevirostris* (ACM/C3-Aj)	0	1	1
*D. fontani* (ACM/C3-Ae)	0	0	1
*H. crusafonti* (TF)	0	0	1
*H. crusafonti* (CP1)	0	2	2
*H. laietanus* (CF)	0	0	1
*H. laietanus* (CLL1)	0	3	1
Total	0	7	8
Total (%)	0	46.67	53.33
**Other hominoids from Western Eurasia**			
**Taxon (locality)**	**Folivores**	**Frugivores**	**Hard-object feeders**
*G. alpani* (Paşalar)^1^	0	0	1
*H. hungaricus* (Rudabánya)^2^	0	0	2
*O. bambolii* (various locs.)^2^	2	6	1
*Ou. macedoniensis* (various locs.)^2^	0	0	7
Total	2	6	11
Total (%)	10.52	31.57	57.89

See Table 1 for locality abbreviations, and Tables S2 and S3 for further information of the discriminant analyses.

1 No individual data available; based on the average value (N = 18) reported in ref. [12].

2 Individual data taken from ref. [10].

## Discussion

### Miocene Hard-Object Feeders

Only the single individual of *P. catalaunicus* fits well with extant HOF, as shown by its high pitting incidence and broad scratches, thus resembling *L*. *albigena* and, especially, *P*. *pygmaeus*. The latter resemble other extant apes in preferring ripe fruits [2], but display thicker enamel as an adaptation to consume harder or unripe fruits, especially as fallback foods [37]. *L*. *albigena* is also a thick-enameled HOF that consumes fleshy fruits but seasonally forages on hard, brittle objects such as nuts and seeds [85], [86]. Although the small sample size precludes a definitive conclusion, our results are consistent with *P. catalaunicus* being a HOF, as previously suggested based on its relatively thick enamel [8] and further confirmed by our multivariate analyses.

Our results also confirm previous inferences, based on pitting incidence, that *Ou. macedoniensis* was a hard-object specialist [10], [14] and that *G. alpani* consumed hard fruits at least as often as orangutans [12], [13]. These taxa are very similar in microwear features to one another, but differ from *P. catalaunicus* and extant HOF by displaying narrower microwear features. This condition of *P. catalaunicus* is more consistent with being a HOF, which compared to FMF and FOL have wider microwear striations (due to higher occlusal forces) [50] as well as larger pits (due to the higher amount of grit routinely ingested by these taxa) [53], [87]–[89]. Differences in microwear feature size among extinct HOF might reflect their divergent habitats and ecological niches. Thus, the orthograde bodyplan with adaptations for vertical climbing and above-branch palmigrady of *P. catalaunicus* [4], [29], [90], [91] suggests a strong arboreal commitment, as in orangutans and the other extant HOF (*L*. *albigena* and *C*. *apella*) [92], [93]. In contrast, the postcranials of *G. alpani* suggest a pronograde bodyplan more suitable for semi-terrestrial quadrupedalism [5], [94], [95]. Similarly, the relatively large body mass [21] and open, pure C_3_ environments inferred for *Ou. macedoniensis* based on the associated fauna [96], [97] also agree with a semi-terrestrial locomotion. Microwear differences between *P. catalaunicus* and other extinct HOF might be thus attributable to differences in the mechanical properties of the food items found in the canopy as opposed to closer to the ground [15], [88], in agreement with previous microwear inferences of a diet primarily based on hard, abrasive items (roots, tubers and/or grasses) for *Ou. macedoniensis* [14]. Alternatively, microwear differences among these taxa might be related to differences in the content of exogenous grit versus phytoliths in the foods consumed, as previously shown for incisor microwear [73]. Abrasive dust particles are more abundant but smaller on average in dry compared to humid environments [88], suggesting that the higher pitting incidences and lower striation breadths of *G. alpani* and *Ou. macedoniensis* might merely reflect their more open and drier habitats compared to both *P. catalaunicus* and extant HOF. Thus, these former taxa might have been predominantly (semi-)terrestrial hard-object feeders, whereas *P. catalaunicus* is best interpreted as an arboreal hard-fruit forager.

### Miocene Soft-Fruit Eaters

Among the taxa analyzed, only *O*. *bambolii* is best interpreted as a soft frugivore, thus contradicting previous interpretations of a specialized folivorous diet [9], [10]. Both pitting percentage and striation breadth suggest some dietary diversity (a few individuals show closer microwear resemblances to either FOL and HOF), as further confirmed by the multivariate analyses. *Cebus nigrivittatus*—a mainly frugivorous primate that further consumes a significant proportion of leaves [98]—might be a good analogue of *O. bambolii*, as shown by their almost identical pitting percentages (16.20% and 16.96%). However, the wide range of pitting incidence displayed by *O. bambolii* does point towards a high dietary flexibility, sporadically including leaves and hard fruits alike, instead of specialized folivory. Although the marked development of molar shearing crests in this taxon was interpreted as a folivorous specialization [9], its pronounced dental relief (with multiple accessory cusps, crests and cingula) more closely resembles that of omnivorous suoids rather than folivores [25], [99], in agreement with our microwear results and its moderately thick enamel [100]. *O*. *bambolii* is thus best interpreted as an eclectic FMF, more clearly relying on soft fruits than other hominoids from Western Eurasia, but further exploiting other resources, possibly due to the trophic restrictions characteristic of insular environments [25], [99].

### Miocene Mixed Soft/Hard-Fruit Feeders

The remaining hominoids from Western Eurasia do not comfortably fall into any of the extant dietary categories, being somewhat intermediate between FMF and HOF. On average, *A. brevirostris* and *H. laietanus* are classified as FMF, whereas *D. fontani*, *H. crusafonti* and *H. hungaricus* display closer affinities with HOF. Individual values, however, are classified as both FMF and HOF, except for the two individuals of *H. hungaricus* and the single specimen of *D. fontani*. The restricted samples for the latter taxa do not enable to ascertain whether they significantly differed from *H. laietanus* and/or *H. crusafonti*. However, when pitting incidence and striation breadth are considered simultaneously, most of the analyzed individuals are intermediate between extant FMF and HOF, not overlapping with other extinct hominoids here interpreted as HOF. Our analyses therefore suggest that the diet of these taxa might have been intermediate between FMF and HOF (by including both soft and hard fruits to a large proportion), thus lacking an appropriate analog among the comparative sample. Such interpretation differs from previous inferences, based on microwear and shearing crest analyses [9]–[11], of a mainly frugivorous diet for *Hispanopithecus* species, and clearly discounts a folivorous diet for *H. laietanus* based on buccal microwear [101].

In these taxa, emphasis on hard-object feeding might have varied depending on the species and/or fluctuated depending on environments. Available samples are too small to adequately test whether hard-food items were consumed as fallback foods, depending on seasonal factors influencing resource availability [102], or whether these species displayed an eclectic foraging strategy on a regular, non-seasonal basis. For species of *Hispanopithecus*, paleoenvironmental reconstructions tend to favor the former hypothesis, since these taxa inhabited humid and subtropical to warm-temperate environments [59], [75], [103], [104]. Such environments would have provided soft fruits at least during part of the year. However, the linear enamel hypoplasias frequently displayed by species of *Hispanopithecus* [105], [106] indicate repeated episodes of malnutrition due to seasonal fluctuations in resource abundance (due to fruiting cycles) [103], [106], [107], thus suggesting that they might have consumed hard-food items as fallback foods during the unfavorable season. Whereas postcranial remains are unknown for *A. brevirostris* and very restricted for *D. fontani* [4], the more complete postcranial remains of *H. laietanus* and *H. hungaricus* clearly evidence a high arboreal commitment and more derived suspensory adaptations than in *P. catalaunicus* [4], [77], [90], [95], [108]–[110]. Species of *Hispanopithecus* are thus best interpreted as arboreal feeders, with their enhanced suspensory capabilities enabling a more efficient foraging on terminal branches [77], [109].

### Evolutionary Implications

Although hominoids are first recorded in Eurasia ca. 17 Ma, coinciding with the beginning of the Miocene Climatic Optimum [3], [4], [111], additional hominoid dispersal events between Africa and Europe probably took place afterwards [3], [4], [6]. Kenyapithecines [4], [112] extended their range into Eurasia before 14 Ma—*G. alpani* and *Kenyapithecus kizili* [2], [17]—and apparently gave rise to the Eurasian hominoid radiation [3], [4], [30]. Besides their likely semi-terrestrial locomotion [94], the dispersal of kenyapithecines was apparently facilitated by their HOF adaptations [4], [7], [8], enabling them to occupy subtropical and highly seasonal, single-canopied woodland/forest with abundant ground vegetation and more open areas [2]. This agrees with our results, suggesting that *G. alpani* mainly relied on hard food items. Available evidence suggests that kenyapithecines rapidly spread throughout Eurasia and vicariantly diversified into different clades [4], [113], with pongines being recorded in Asia and dryopithecines in the Iberian Peninsula by ca. 12.5 Ma [4].

The European dryopithecines are alternatively interpreted as stem hominids [29], [30], hominines [5], [6], or the sister-taxon of Asian pongines [4], [114]. Between 12.5 and 7 Ma, and despite a climatic trend towards cooling and increased seasonality [4], [115], they experienced an adaptive radiation from both taxonomic and ecological viewpoints [4], [6]. This radiation, including the acquisition of new locomotor and dietary adaptations, was probably related to the new selection pressures posed by the different biotopes present in Europe during the Middle and Late Miocene, coupled with regional paleoenvironmental differences and changes through time [4], [6]. Both the habitat and diet of *G. alpani* from Turkey most closely resemble those of their Middle Miocene relatives from Africa [2], [113], [116]. In contrast, the habitats encountered by subsequent hominoids in both NE Spain and Central Europe were more humid, less seasonal, and more densely-forested [116]–[118]. Our results suggest that *P*. *catalaunicus* retained the ancestral HOF strategy while specializing for arboreal foraging. In contrast, *D. fontani* and *A. brevirostris* apparently displayed a somewhat more frugivorous diet (albeit with some sclerocarpic component), which might be interpreted as an early adaptive response to the new environmental conditions. Competitive exclusion coupled with increased environmental heterogeneity of the plant communities—which favors increased paleobiodiversity by multiplying the number of available ecological niches [119]—would have played a role in the dietary diversification of dryopithecines.

The dietary diversification of hominoids in Western Eurasia was further accentuated during the Late Miocene—as shown by the mixed soft-hard frugivorous condition of *Hispanopithecus*, the more frugivorous but versatile diet of *O. bambolii*, and the specialized hard-object feeding of *Ou. macedoniensis*. The adaptive trend towards increased soft frugivory in *Hispanopithecus* is at odds with concomitant climatic changes toward increased seasonality and lower temperatures. These environmental changes prompted the substitution of evergreen by deciduous trees [120] as well as the fragmentation of habitats suitable for frugivorous hominoids [103]. For some time, *Hispanopithecus* overcame such a paleoenvironmental deterioration thanks to new locomotor adaptations—presumably enabling a more efficient foraging on the canopy [77], [109]—instead of exploiting a greater proportion of leafy material and/or foraging on the ground.

The extinction of hominoids in Europe was ultimately related to an increase in environmental uniformity and the resulting loss of suitable habitats [96]. In Western and Central Europe, it has been related to the substitution of (sub)tropical plants by deciduous trees [120]. At least in the Vallès-Penedès, however, this process was gradual, implying the generation of mosaic environments in which (sub)tropical elements became progressively restricted to lowland humid areas [4], [103]. *Hispanopithecus* probably had to seasonally recourse to hard food items as fallback foods, until the reduction of its preferred habitat ultimately caused its extinction in Spain and elsewhere in Europe [4], [96], [103].

In Eastern Europe, *Ou. macedoniensis* survived longer than *Hispanopithecus* (ca. 8.0-7.5 vs. 9.5 Ma), probably thanks to a specialized terrestrial HOF diet adapted to open and arid habitats with a predominantly herbaceous vegetation with abundant C_3_ grasses, bushes and herbs [96]. Although its extinction did not coincide with any major climatic shift, it was similarly related to strong seasonal variations [97]. The paleoenvironmental changes leading to the extinction of *Ou. macedoniensis* were apparently opposite to those experienced by *Hispanopithecus*, since in Eastern Europe bushy vegetation expanded over areas previously occupied by grasslands [94], whereas in Western and Central Europe it was rather accompanied by the substitution of (sub)tropical plants by deciduous trees [120].

*O. bambolii* survived much longer in the Tusco-Sardinian insular ecosystems by displaying a more frugivorous but highly versatile diet, allowing it to opportunistically exploit more fibrous plant materials and harder fruits alike. Its extinction does not offer much insight with regard to that of other apes from mainland Europe, since it is not related to any environmental change [121], but rather to the connection of its insular habitat to the mainland ca. 7 Ma—and the substitution of the endemic associated fauna [26], [121].

## Conclusions

Contrary to previous interpretations, our microwear analyses show that leaves and stems were not a primary dietary component for any hominoid from Western Eurasia, which are interpreted instead as frugivores (*O. bambolii*) and/or hard-object feeders (e.g., *P. catalaunicus*). Whereas some of the studied taxa fall comfortably within these two dietary categories, many of them (such as *Hispanopithecus* species) seem to be intermediate, suggesting that they have no extant dietary analog in the comparative sample.

From a evolutionary perspective, our results indicate that hominoids from Western Eurasia experienced a progressive dietary diversification between 14 and 7 Ma, from the presumably ancestral condition of (semi-)terrestrial hard-object feeding shown by *G. alpani*. In Western and Central Europe, this diversification might have been triggered by changes in habitat structure (more densely-forested environments), coupled with competitive exclusion and new locomotor adaptations related to arboreal feeding (as shown by *P. catalaunicus*). Other taxa from this area (especially the species of *Hispanopithecus*) apparently combined soft and hard fruits in their diets. The high behavioral plasticity of extant great apes allows them to survive in front of a marked environmental instability (resulting in spatial/temporal uncertainty of preferred fruit resources) [122], [123]. Similarly, the suspensory adaptations of *Hispanopithecus* species (enabling a more efficient foraging on terminal branches), coupled with the exploitation of harder food items during the unfavorable season, might have allowed them to temporarily overcome the progressive environmental deterioration. Ultimately, however, the restriction and fragmentation of their preferred habitats would have led to their extinction from Western and Central Europe. In contrast, *Ou. macedoniensis* survived longer in the more open and arid landscapes of Eastern Europe by displaying a more terrestrial trophic niche based on hard food items, whereas *O*. *bambolii* persisted even longer in the Tusco-Sardinian Paleobioprovince by displaying a versatile frugivorous diet, until its insular ecosystem was connected to the mainland.

The failure by any of these taxa to adapt to folivory in the face of environmental changes towards increased seasonality might be attributable to their specialized (although diverging) trophic niches. The contrasting environmental changes experienced by the respective habitats of *Hispanopithecus* (more deciduous and open forests) and *Ou. macedoniensis* (less open and more bushy habitats), coupled with their strikingly divergent trophic niches, suggest that great-ape vulnerability to environmental change is not attributable to a frugivorous bias per se [122], but rather to the adaptation to whatever hyperspecialized trophic niche.

## Supporting Information

Figure S1 **Bivariate plots of microwear feature breadth vs. pitting incidence.** (A) Striation breadth and (**B**) pit breadth vs. pitting incidence based on individual values reported in Table 2. See Figure 2 for the equivalent plots based on mean species/locality values reported in Table 1. Abbreviations as in Figure 2 (note that symbols and colors are different to show variability within fossil species).
(TIF)

Figure S2 **Results of the cluster analysis based on dental microwear features for individual values.** Note that symbols and colors are different to those of Figure 2 to show variability within fossil species. See Figure 3 for the results based on mean species/locality data.
(TIF)

Table S1 **Sample sizes for the studied extinct species.**
(DOCX)

Table S2 **Results of the CVA based on microwear features.**
(DOCX)

Table S3 **Scores for the two canonical variates in extant and extinct taxa derived by the CVA.**
(DOCX)

Table S4 **Individual results of the discriminant analysis based on the CVA.**
(DOCX)
