# Supplementary figures and images for: Dietary Specialization during the Evolution of Western Eurasian Hominoids and the Extinction of European Great Apes
Source: PLoS One. 2014 May 21;9(5):e97442. doi: 10.1371/journal.pone.0097442 (PMC4029579; doi:10.1371/journal.pone.0097442)

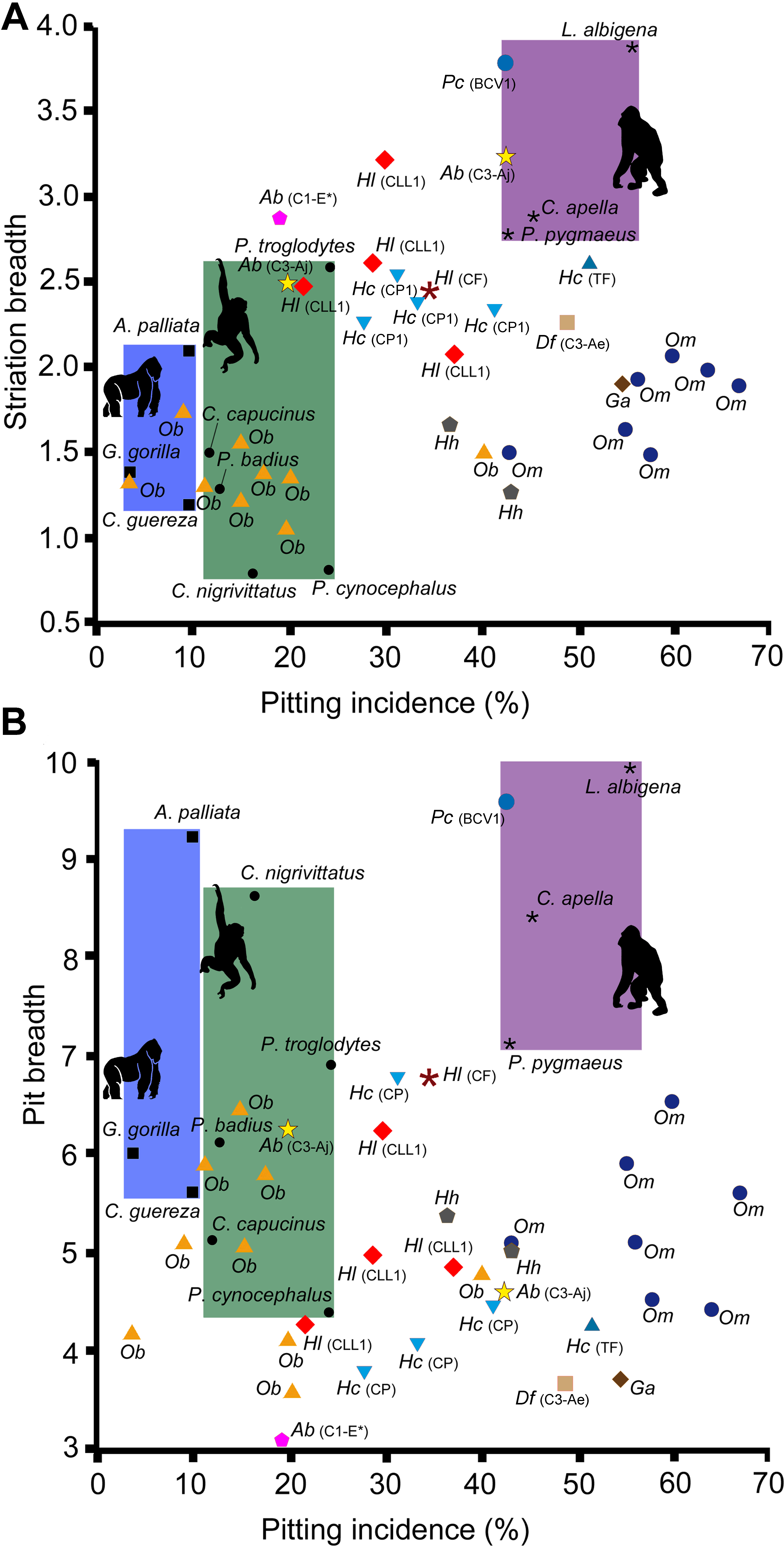

Supplement: Figure S1 — Bivariate plots of microwear feature breadth vs. pitting incidence. (A) Striation breadth and (B) pit breadth vs. pitting incidence based on individual values reported in Table 2. See Figure 2 for the equivalent plots based on mean species/locality values reported in Table 1. Abbreviations as in Figure 2 (note that symbols and colors are different to show variability within fossil species). (TIF) [file pone.0097442.s001.tif]

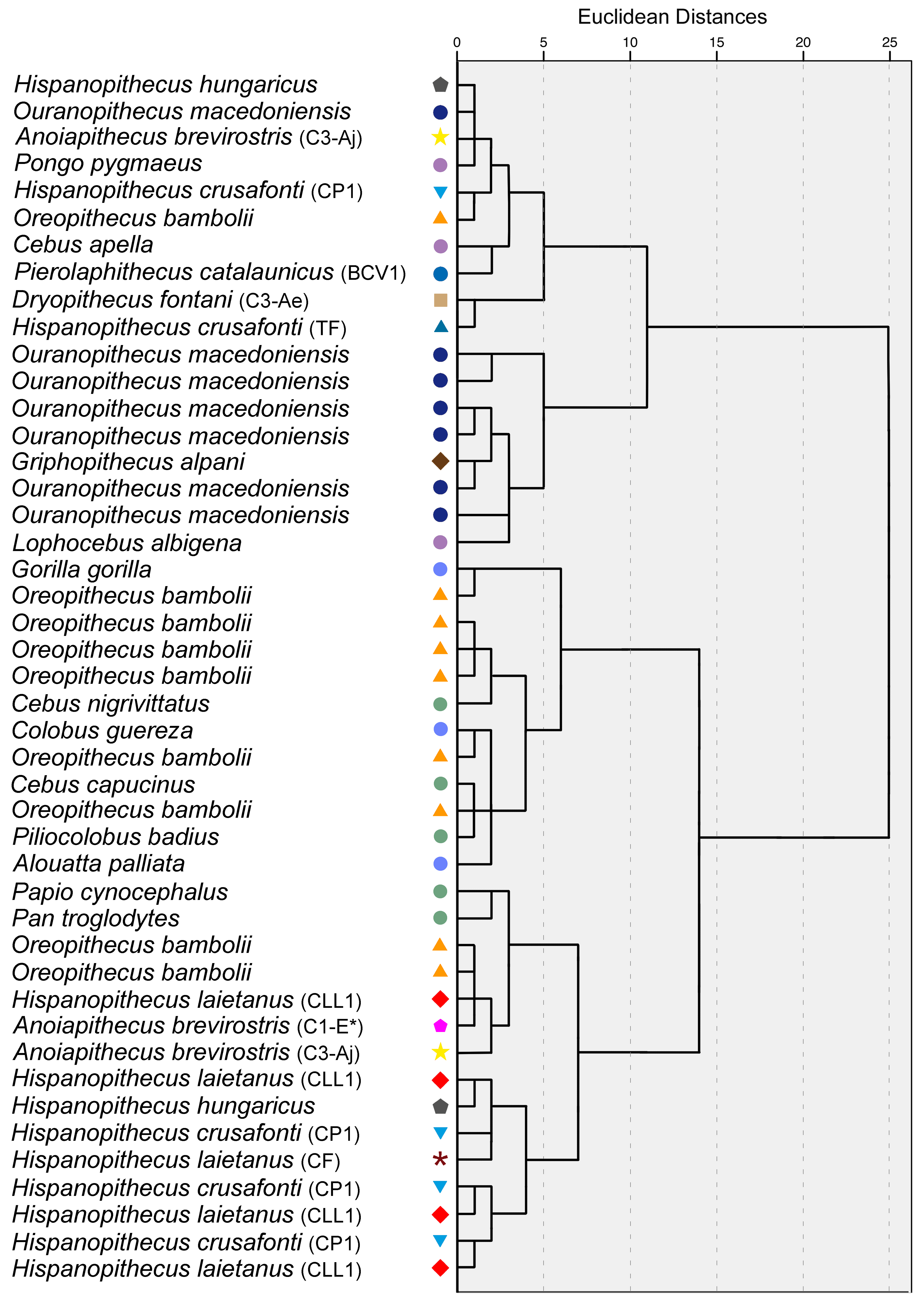

Supplement: Figure S2 — Results of the cluster analysis based on dental microwear features for individual values. Note that symbols and colors are different to those of Figure 2 to show variability within fossil species. See Figure 3 for the results based on mean species/locality data. (TIF) [file pone.0097442.s002.tif]
