# Supplementary material for: Dietary Specialization during the Evolution of Western Eurasian Hominoids and the Extinction of European Great Apes
Source: PLoS One. 2014 May 21;9(5):e97442. doi: 10.1371/journal.pone.0097442 (PMC4029579; doi:10.1371/journal.pone.0097442)
Supplement: Table S1 — Sample sizes for the studied extinct species. (DOCX) [file pone.0097442.s003.docx]

**Table S1. Sample sizes for the studied extinct species.**

| **Taxon** | **Locality** | **Age (Ma)** | **N** |
| --- | --- | --- | --- |
| *Pierolapithecus catalaunicus* | Abocador de Can Mata/Barran de Can Vila 1 | 11.93 | 1 |
| *Anoiapithecus brevirostris* | Abocador de Can Mata/C1-E* | 12.3–12.2 | 1 |
| *Anoiapithecus brevirostris* | Abocador de Can Mata/C3-Aj | 11.94 | 2 |
| *Anoiapithecus brevirostris* | average | 12.3–11.94 | 3 |
| *Driopithecus fontani* | Abocador de Can Mata/C3-Ae | 11.85 | 1 |
| *Hispanopithecus crusafonti* | Teuleria del Firal | 10.4–10.0 | 1 |
| *Hispanopithecus crusafonti* | Can Poncic 1 | 10.4–10.0 | 4 |
| *Hispanopithecus crusafonti* | average | 10.4–10.0 | 5 |
| *Hispanopithecus laietanus* | Can Feu | 10.0–9.7 | 1 |
| *Hispanopithecus laietanus* | Can Llobateres 1 | 9.72 | 4 |
| *Hispanopithecus laietanus* | average | 10.0–9.7 | 5 |
| *Gripopithecus alpani***^1^** | Paşalar | 14.9–13.7 | 18 |
| *Hispanopithecus hungaricus***^1^** | Rudabánya | 10.0–9.8 | 2 |
| *Oreopithecus bambolii***^1^** | Baccinello, Monte Bamboli, Ribolla | 8.3–6.7 | 9 |
| *Ouranopithecus macedoniensis***^1^** | Ravin de la Pluie, Xirochori, Nikiti | 9.7–9.0 | 7 |

Abbreviations: Ma, million years ago; N, sample size.

**^1^** Data taken from refs. [10, 12].
