# Supplementary material for: Dietary Specialization during the Evolution of Western Eurasian Hominoids and the Extinction of European Great Apes
Source: PLoS One. 2014 May 21;9(5):e97442. doi: 10.1371/journal.pone.0097442 (PMC4029579; doi:10.1371/journal.pone.0097442)
Supplement: Table S2 — Results of the CVA based on microwear features. (DOCX) [file pone.0097442.s004.docx]

**Table S2. Results of the CVA based on microwear features.**

| **CV** | **Eigenvalue** | **Variance (%)** | | | **Cumulative variance (%)** | **Canonical correlation** |
| --- | --- | --- | --- | --- | --- | --- |
| CV1 | 10.355 | 96.7 | | | 96.7 | 0.955 |
| CV2 | 0.348 | 3.3 | | | 100.0 | 0.508 |
|  | **Standardized coefficients of the CV** | | | | | |
| **CV** | **Pitting percentage** | | | **Pit breadth** | | **Scratch breadth** |
| CV1 | 1.045 | | | -0.093 | | -0.055 |
| CV2 | -0.442 | | | 0.346 | | 0.940 |
|  | **Non-standardized coefficients of CV** | | | | | |
| **CV** | **Pitting percentage** | **Pit breadth** | | | **Scratch breadth** | **Constant** |
| CV1 | 0.183 | -0.055 | | | -0.086 | -3,673 |
| CV2 | -0.077 | 0.206 | | | 1.454 | -2,477 |
|  | **Scores for group centroids** | | | | | |
| **CV** | **Folivores** | | **Frugivores** | | | **Hard-object feeders** |
| CV1 | -2.800 | | -0.896 | | | 4.294 |
| CV2 | 0.642 | | -0.526 | | | 0.235 |

See canonical scores for extant and extinct taxa in Table S3, and dietary category prediction results for extinct taxa, based on the discriminant analysis, in Table 4.

Abbreviations: CV, canonical variate.
