# Supplementary material for: Dietary Specialization during the Evolution of Western Eurasian Hominoids and the Extinction of European Great Apes
Source: PLoS One. 2014 May 21;9(5):e97442. doi: 10.1371/journal.pone.0097442 (PMC4029579; doi:10.1371/journal.pone.0097442)
Supplement: Table S3 — Scores for the two canonical variates in extant and extinct taxa derived by the CVA. (DOCX) [file pone.0097442.s005.docx]

**Table S3. Scores for the two canonical variates in extant and extinct taxa derived by the CVA.**

| **Taxon** | **CV1** | **CV2** | **Diet** |
| --- | --- | --- | --- |
| **Extant species (N)** |  |  |  |
| *Gorilla gorilla* (10) | -3.5025 | 0.5320 | FOL |
| *Alouatta palliata*(10) | -2.5865 | 1.7217 | FOL |
| *Colobus guereza* (10) | -2.3107 | -0.3289 | FOL |
| *Cebus capucinus* (10) | -1.9429 | -0.1507 | FMF |
| *Piliocolobus badius* (10) | -1.8163 | -0.3050 | FMF |
| *Cebus nigrivittatus*(10) | -1.2528 | -0.7953 | FMF |
| *Papio cynocephalus* (16) | 0.3803 | -2.2314 | FMF |
| *Pan troglodytes* (10) | 0.1504 | 0.8517 | FMF |
| *Pongo pygmaeus* (10) | 3.4703 | -0.2331 | HOF |
| *Cebus apella* (10) | 3.8656 | -0.0210 | HOF |
| *Lophocebus albigena* (10) | 5.5450 | 0.9601 | HOF |
| **Hominoids from the Iberian Peninsula (locality)** |  |  |  |
| *Pierolaphithecus catalaunicus* (ACM/BCV1) | 3.2480 | 1.7195 |  |
| *Anoiapithecus brevirostris* (ACM/C1-E*) | -0.5694 | 0.8354 |  |
| *Anoiapithecus brevirostris* (ACM/C3-Aj) | 1.4872 | 0.3859 |  |
| *Anoiapithecus brevirostris* (average) | 0.802 | 0.536 |  |
| *Dryopithecus fontani* (ACM/C3-Ae) | 4.8617 | -2.2305 |  |
| *Hispanopithecus crusafonti* (TF) | 5.2717 | -1.8018 |  |
| *Hispanopithecus crusafonti* (CP1) | 1.9758 | -0.6343 |  |
| *Hispanopithecus crusafonti* (average) | 2.635 | -0.868 |  |
| *Hispanopithecus laietanus* (CF) | 2.0758 | -0.2136 |  |
| *Hispanopithecus laietanus* (CLL1) | 1.1902 | 0.0648 |  |
| *Hispanopithecus laietanus* (average) | 1.368 | 0.006 |  |
| **Other hominoids from Western Eurasia (locality)** |  |  |  |
| *Griphopithecus alpani* (Paşalar) | 5.9488 | -3.1794 |  |
| *Hispanopithecus hungaricus* (Rudabánya) | 3.1923 | -2.3624 |  |
| *Oreopithecus bambolii* (Baccinello, Monte Bamboli, Ribolla) | -0.9630 | -0.7821 |  |
| *Ouranopithecus macedoniensis* (Ravin de la Pluie, Xirochori, Nikiti) | 6.4366 | -3.2653 |  |

See Table S2 for further details on the CVA results.

Abbreviations: CV, canonical variate; FOL, folivores; FMF, frugivores/mixed-feeders; HOF, hard-object feeders.

Mean extant species data were taken from refs. [50, 71], whereas mean species/locality data for extinct taxa from the Iberian Peninsula are reported in Table 1. Mean data for other hominoids from Western Eurasia were taken from refs. [10, 12]. See ref. [34] for information regarding the extant dietary categories employed to define groups a priori.
