# Supplementary material for: Dietary Specialization during the Evolution of Western Eurasian Hominoids and the Extinction of European Great Apes
Source: PLoS One. 2014 May 21;9(5):e97442. doi: 10.1371/journal.pone.0097442 (PMC4029579; doi:10.1371/journal.pone.0097442)
Supplement: Table S4 — Individual results of the discriminant analysis based on the CVA. (DOCX) [file pone.0097442.s006.docx]

**Table S4. Individual results of the discriminant analysis based on the CVA.**

| **Taxon** | **Locality** | **Catalog No.** | **1st group** | **p** | **D^2^** | **2nd group** | **D^2^** |
| --- | --- | --- | --- | --- | --- | --- | --- |
| *P. catalaunicus* | ACM/BCV1 | IPS21350 | HOF | 0.192 | 3.296 | FMF | 22.219 |
| *A. brevirostris* | ACM/C1-E* | IPS35027 | FMF | 0.375 | 1.961 | FOL | 5.012 |
| *A. brevirostris* | ACM/C3-Aj | IPS41712 | FMF | 0.350 | 2.101 | FOL | 5.016 |
| *A. brevirostris* | ACM/C3-Aj | IPS43000 | HOF | 0.712 | 0.679 | FMF | 19.922 |
| *D. fontani* | ACM/C3-Ae | IPS35026 | HOF | 0.041 | 6.403 | FMF | 36.059 |
| *H. crusafonti* | TF | MGSB25314 | HOF | 0.078 | 5.107 | FMF | 39.672 |
| *H. crusafonti* | CP1 | CP1815 | HOF | 0.043 | 6.293 | FMF | 8.430 |
| *H. crusafonti* | CP1 | CP1818 | FMF | 0.047 | 6.124 | HOF | 7.956 |
| *H. crusafonti* | CP1 | CP1820 | FMF | 0.160 | 3.670 | HOF | 11.395 |
| *H. crusafonti* | CP1 | CP1821 | HOF | 0.187 | 3.359 | FMF | 19.293 |
| *H. laietanus* | CF | IPS34753 | HOF | 0.077 | 5.120 | FMF | 8.931 |
| *H. laietanus* | CLL1 | IPS1763 | FMF | 0.540 | 1.231 | FOL | 7.104 |
| *H. laietanus* | CLL1 | IPS1788 | FMF | 0.113 | 4.356 | HOF | 10.251 |
| *H. laietanus* | CLL1 | IPS1797 | HOF | 0.077 | 5.137 | FMF | 13.339 |
| *H. laietanus* | CLL1 | IPS1800 | FMF | 0.028 | 7.159 | HOF | 10.711 |
| *G.alpani***^1^** | Paşalar | — | HOF | 0.001 | 14.400 | FMF | 53.895 |
| *H.hungaricus***^2^** | Rudabánya | RUD 77 | HOF | 0.006 | 10.275 | FMF | 27.825 |
| *H. hungaricus***^2^** | Rudabánya | RUD 141 | HOF | 0.029 | 7.086 | FMF | 13.778 |
| *O. bambolii***^2^** | Baccinello | BAC 60 | FMF | 0.510 | 1.345 | FOL | 1.425 |
| *O. bambolii***^2^** | Baccinello | BAC 66 | FMF | 0.558 | 1.166 | FOL | 10.645 |
| *O. bambolii***^2^** | Baccinello | BAC 69 | FMF | 0.788 | 0.477 | FOL | 2.423 |
| *O. bambolii***^2^** | Baccinello | BAC 198 | FMF | 0.882 | 0.251 | FOL | 4.767 |
| *O. bambolii***^2^** | Baccinello | BAC 104 | FMF | 0.989 | 0.022 | FOL | 5.300 |
| *O. bambolii***^2^** | Monte Bamboli | MONT 23 | FMF | 0.436 | 1.660 | FOL | 11.509 |
| *O. bambolii***^2^** | Ribolla | RIB 18 | HOF | 0.016 | 8.226 | FMF | 21.430 |
| *O. bambolii***^2^** | Ribolla | RIB 21 | FOL | 0.905 | 0.200 | FMF | 3.207 |
| *O. bambolii***^2^** | Ribolla | RIB 22 | FOL | 0,703 | 0.705 | FMF | 6.308 |
| *Ou. macedoniensis***^2^** | Ravin de la Pluie | RPL 54 | HOF | 0.015 | 8.341 | FMF | 26.279 |
| *Ou. macedoniensis***^2^** | Ravin de la Pluie | RPL 55 | HOF | 0.000 | 25.518 | FMF | 81.604 |
| *Ou. macedoniensis***^2^** | Ravin de la Pluie | RPL 75 | HOF | 0.000 | 22.028 | FMF | 66.499 |
| *Ou. macedoniensis***^2^** | Ravin de la Pluie | RPL 76 | HOF | 0.001 | 14.439 | FMF | 57.099 |
| *Ou. macedoniensis***^2^** | Ravin de la Pluie | RPL 128 | HOF | 0.000 | 15.538 | FMF | 64.509 |
| *Ou. macedoniensis***^2^** | Nikiti | NKT 21 | HOF | 0.000 | 31.063 | FMF | 92.295 |
| *Ou. macedoniensis***^2^** | Xirochi | XIR 1 | HOF | 0.001 | 14.473 | FMF | 54.101 |

See Table S2 for further details on the discriminant analysis, and Table 3 for summary results per species/locality.

Abbreviations: D^2^, Squared Mahalanobis distance; FOL, folivores; FMF, frugivores; HOF, Hard-object feeders.

**^1^** No individual data available; based on the average value (N=18) reported in Supplementary ref. [12].

**^2^** Data taken from Supplementary ref. [10].
